# A case series of thoracic dynamic contrast-enhanced MR lymphangiography: technique and applications

**DOI:** 10.1259/bjrcr.20200026

**Published:** 2020-04-30

**Authors:** Cheng Xie, Catriona Stoddart, Anthony McIntyre, Victoria StNoble, Heiko Peschl, Rachel Benamore

**Affiliations:** 1 Department of Radiology, Churchill Hospital, Oxford University Hospital Trust, Old Road, Headington, Oxford, OX3 7LE

## Abstract

Dynamic contrast-enhanced magnetic resonance lymphangiography is a radiation-free, high spatial resolution technique which is increasingly used to evaluate thoracic lymphatic disorders and for pre-procedural planning. DCE has the added advantage of allowing dynamic real-time evaluation of lymphatic flow. It can be employed to investigate commonly encountered clinical situations such as recurrent pleural effusions following trauma, thoracic duct injury after thoracic surgery, and exclude diseases and congenital malformations of the thoracic lymphatic system. The imaging procedure and protocol are detailed in this case series to highlight the application of dynamic contrast-enhanced magnetic resonance lymphangiography in everyday practice and its importance to guide surgical planning.

## Introduction

**Figure 1.**
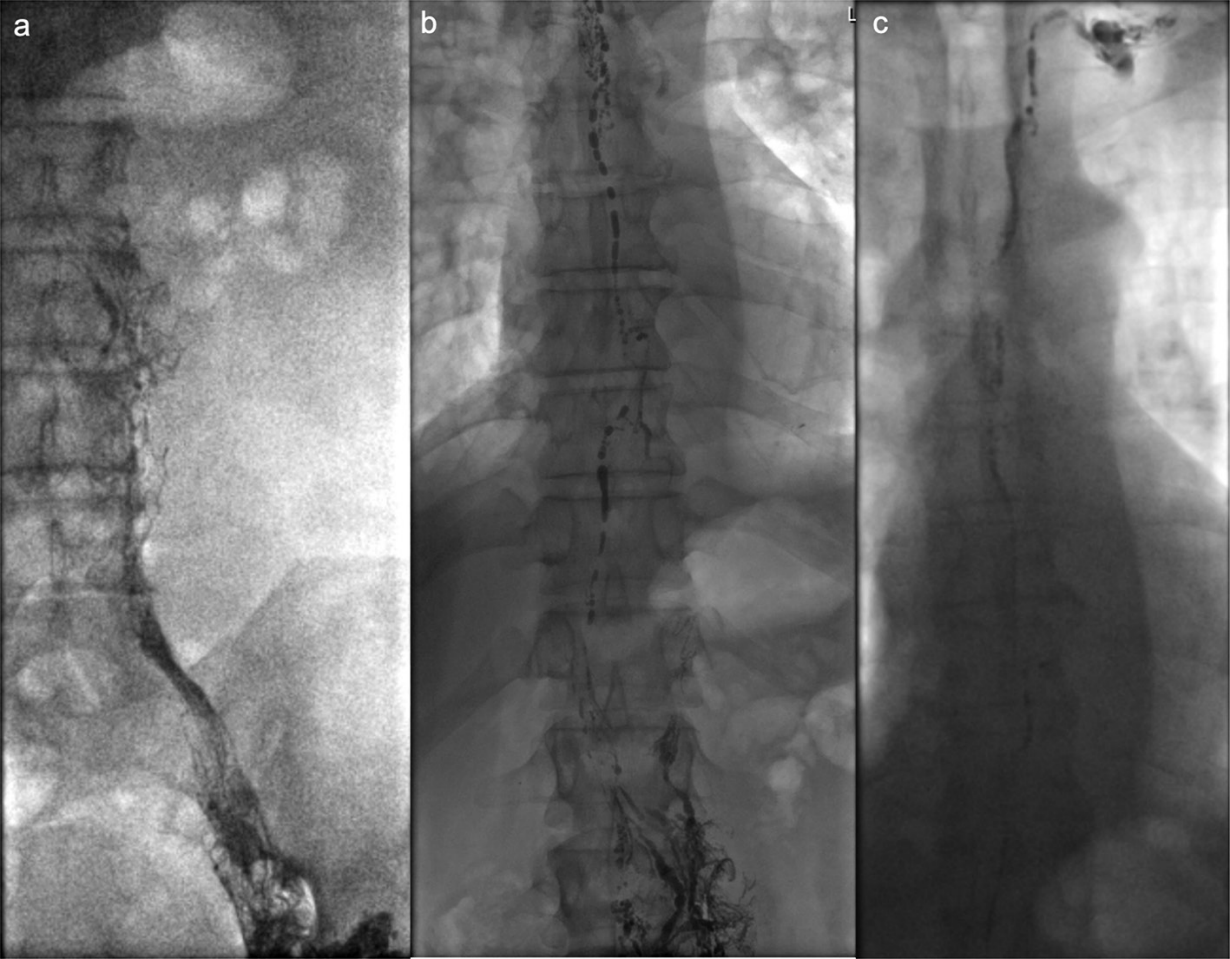
Fluoroscopic lymphangiography with contrast material from the left inguinal lymphatics (Figure 1a) and moving into the central lymphatic system (Figure 1b–c).

**Figure 2.**
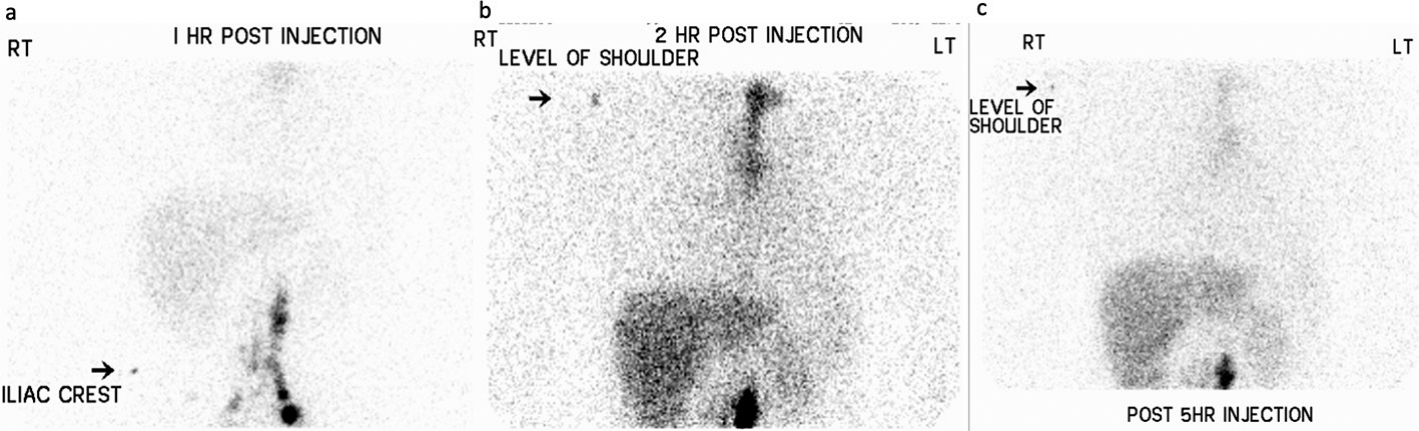
Lymphoscintigraphy of patient with recurrent pleural effusions. Study showed normal nodal uptake in the abdominal para-aortic nodal chains with activity within the thoracic duct between 1 and 2 h (Figure 2a–b). By the end of the study at 5 h, there was no activity in the moderate right pleural effusion (Figure 2c).

The traditional methods of lymphatic imaging include fluoroscopic lymphangiography and lymphoscintigraphy. Fluoroscopic lymphangiography involves tracking and visualising the contrast material as it moves from the cannulated peripheral lymphatic into the central lymphatic system (Figure 1a–c). Lymphoscintigraphy is frequently used to localise sentinel node in breast cancer and can be used to investigate lymphatic disorders.^1^ Both methods incur a radiation dose, and lymphoscintigraphy can take hours to complete (Figure 2a–c). MRI is a simple, and a radiation-free alternative investigation. In this case series, we discuss our experience of the imaging technique and protocol of dynamic contrast-enhanced (DCE) magnetic resonance (MR) lymphangiography to help answer commonly encountered clinical questions.

### Function and anatomy of the lymphatic system

The lymphatic system forms part of the circulatory system. Its primary function is to return excess interstitial fluid (lymph) derived from plasma filtration in tissues to the central venous system. Due to the permeable nature of the peripheral lymphatic vessels, lymph contains pathogens, immune cells and in the gut chylomicrons, which facilitate its role in the immune system and the transportation of absorbed fat.^2^ Valves ensure lymph flows unidirectionally around the body and the contraction of skeletal muscle and arterial pulsation facilitate its movement.^3^

The lymphatic system has peripheral and central components. The peripheral lymphatic ducts from the lower extremities and abdomen join to form the central thoracic duct. Most commonly, this confluence is a single 0.5–2 cm wide saccular dilatation called the cisterna chyli, which lies to the right of the abdominal aorta anterior to the first and second lumbar vertebrae.^4,5^ The cisterna chyli measures 38–45 mm long and continues in the thorax as the thoracic duct after passing through the aortic hiatus of the diaphragm. The thoracic duct travels through the posterior mediastinum to the right of the thoracic aorta. At around the fifth thoracic vertebra, it crosses the midline posterior to the oesophagus and ascends in the superior mediastinum posterior to the left subclavian artery. The thoracic duct drains into the venous system at the junction of the left internal jugular and subclavian veins. The thoracic duct receives lymph from lymphatic ducts draining the lungs, mediastinum, left arm, head and neck. Lymphatic drainage from the right side of the head and neck, right arm and thorax drain into the right lymphatic duct and subsequently the right venous angle.^2^

There is considerable variation in the anatomy of the central lymphatic system. The cisterna chyli can have a right paramedian, central or left paramedian position. It may vary in size and can have an inverted Y, inverted V, rope of pearls or comma shape. In some cases, the confluence of the lower extremity lymphatics forms a plexus rather than a single duct and in 30% of patients the cisterna chyli is absent. Variations of the thoracic duct include drainage into the right venous system (2–3%), bifurcation or trifurcation of a single duct or bilateral ducts (1–1.5%). The confluence with the central venous system can be at the internal jugular vein, subclavian vein, the left venous angle or the brachiocephalic vein.^4–7^

## Imaging technique/protocol

### Ultrasound-guided inguinal lymph node cannulation

A dedicated private ultrasound area should be prepared outside the MRI scanning room. However, if your equipment is MRI compatible, then the patient should be on the scanning table right from the start to avoid unnecessary movement and allow immediate scanning after injection of gadolinium. The patient lies in the supine position, and both groins are assessed using ultrasound for the most accessible lymph node on either side. At this stage, measurement from skin to lymph node should be taken to help select the most appropriate length of needle. A 20- to 25-gauge needle is suitable. Under sterile conditions, and ultrasound guidance, the lymph node is cannulated and the correct position confirmed with a test injection of 1–2 mls saline. 5–10 ml 0.1% gadolinium is then injected slowly in the central medulla of the lymph node. The lymph node may burst and cause pain if the rate of injection is too fast. Other diluted concentrations and injection rates such as 1 ml/3 min have been used.^8^ After injection of the inguinal lymph node on both sides, the patient should be swiftly transferred to the scanner. A phased array surface body coil should be used.

### MRI sequence

**Figure 3.**
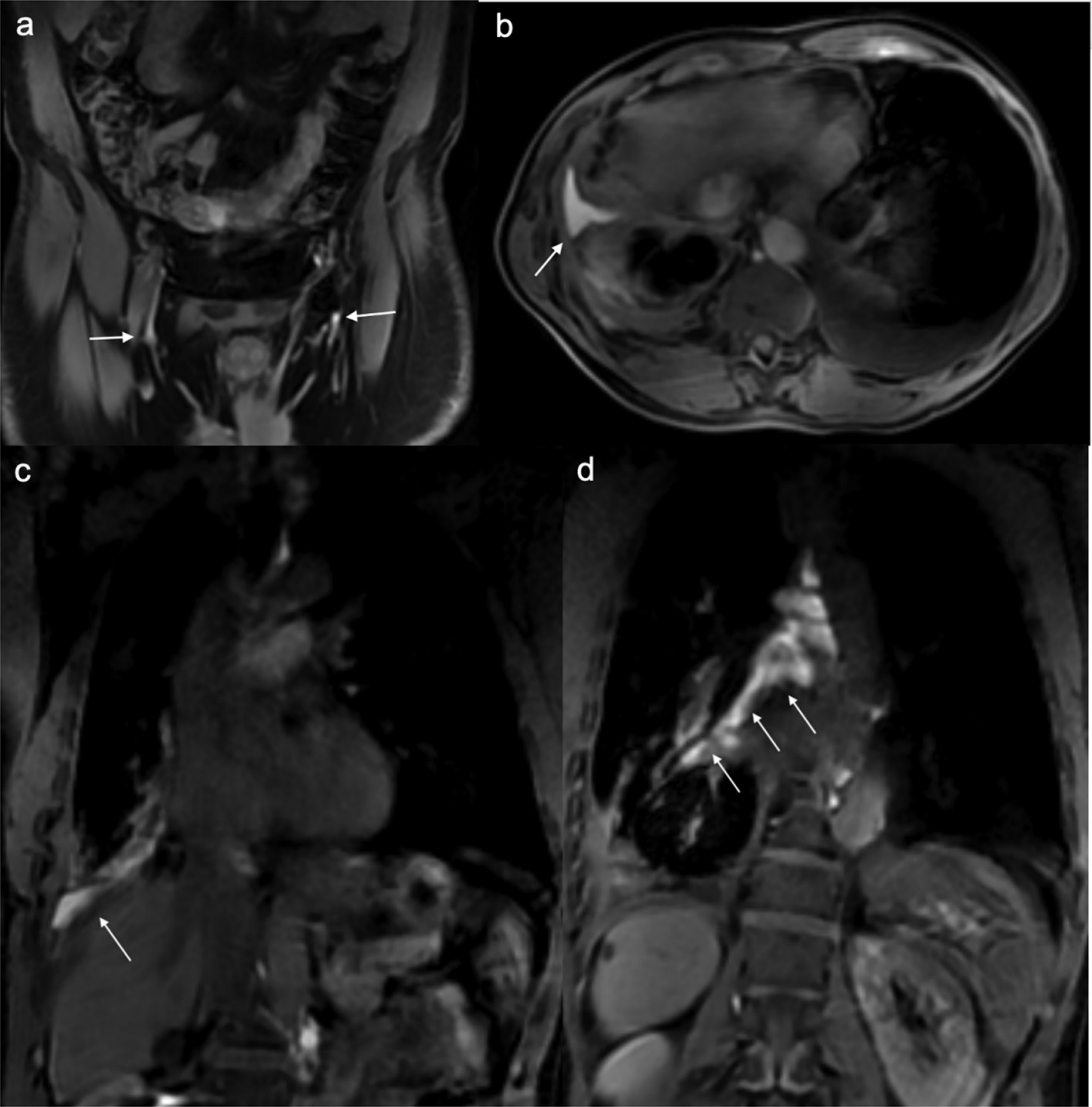
DCE MR lymphangiogram of patient with recurrent pleural effusions. Initial coronal plane *T*_1_ weighted image with fat saturation showed enhancement in the inguinal lymphatic chain bilaterally (Figure 3a, white arrows). Axial and coronary thoracic images showed right pleural effusion with contrast enhancement (Figure 3b–c, white arrow) and could tracking into the right hilum (Figure 3d, white arrows). DCE, dynamic contrast-enhanced; MR, magnetic resonance.

All scans we have performed to date have been on our 3 T scanner (GE Discovery MR750, GE Medical Systems, Milwaukee, WI), although the technique would be compatible with a 1.5 T scanner. Due to the small size of lymphatic ducts (<4 mm in diameter), overlapping thin slice acquisition is important. Generally, the most useful sequence is the breath-hold post-contrast dynamic fat saturated *T*_1_ weighted three-dimensional gradient echo sequence (LAVA – Liver Accelerated Volume Acquisition). Heavily *T*_2_ weighted imaging is also useful to image the fluid filled lymphatics, which is similar to the technique used in biliary MRI. An initial coronal plane helps to provide the anatomy and position of the contrast from the groin up the cisterna chyli (Figure 3a). Subsequent imaging should be performed within 1 min; however, the interval between scans should be adjusted based on the rate of contrast/lymphatic flow. Additional axial and/or sagittal planes should be performed at any suspicious levels of contrast leak. We recommend the presence of a radiologist during the scanning process to work with the radiographer to determine if any additional orthogonal planes are required.

## Results

### Case 1

A 60-year-old male patient who previously had a traumatic road traffic accident and sustained right sided pneumothorax and ribs fractures suffers from recurrent right pleural effusions. He was referred for imaging options to investigate the cause of the recurrent pleural effusions. The patient had a lymphoscintigraphy (Tc-99m-Antimony sulphide colloid). It showed normal nodal uptake in the abdominal para-aortic nodal chains with activity within the thoracic duct between 1 and 2 h. By the end of the study at 5 h, there was no activity in the moderate right pleural effusion and no evidence of Chyle leak (Figure 2a–c). However, a subsequent thoracoscopy and samples confirmed chylothorax. A DCE MR lymphangiogram was performed to locate the site of leak for surgical planning. It showed contrast enhancement of the right pleural effusion (Figure 3b–c). The enhancing fluid could be tracked along the bronchovascular bundles, into the right hilum, and appeared to communicate with the thoracic duct (Figure 3d). The findings were consistent with a lymphatic leak into the right pleural effusion, from the thoracic duct. The patient subsequently had successful right thoracotomy and clipping of thoracic duct.

### Case 2

**Figure 4.**
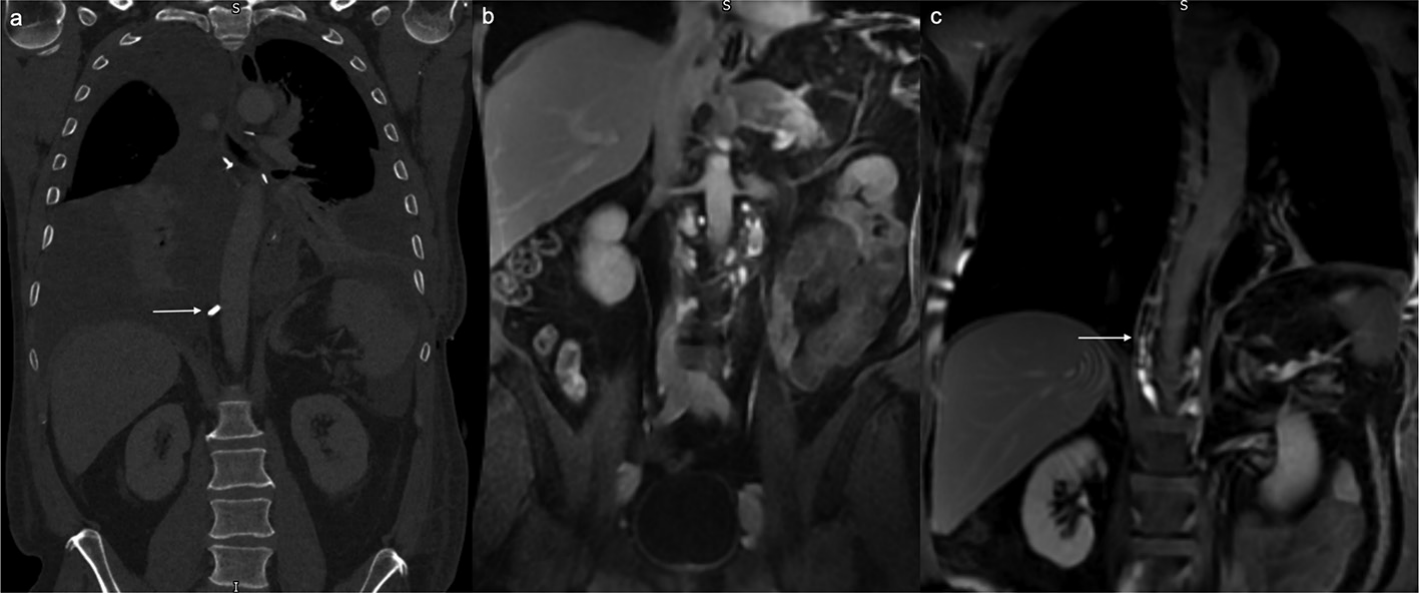
Patient with bilateral pleural effusions post-oesophagectomy on coronal CT with thoracic duct ligation clip (Figure 4a, white arrow). There was progressive enhancement of the abdominal lymphatics, cisterna chyli and inferior thoracic duct up to the level of T11-12 (Figure 4b–c, white arrow), which corresponds to the level of thoracic duct ligation clip on the coronal CT. Contrast did not extend cranially on delayed imaging, in keeping with a ligated thoracic duct.

A 58-year-old male patient had recent oesophagectomy due to oesophageal malignancy. He had bilateral pleural effusions on the post-operative CT (Figure 4a), and went on to have DCE MR lymphangiogram to assess the integrity of the thoracic duct. The coronal *T*_1_ weighted images with fat saturation showed progressive enhancement of the abdominal lymphatics, cisterna chyli and inferior thoracic duct up to the level of T11-12 (Figure 4b–c). This corresponded to the position of the metallic clips seen on the coronal CT and represent the position of thoracic duct ligation. There was no enhancement proximal to this level and no lymphatic leak into the pleural effusion.

### Case 3

**Figure 5.**
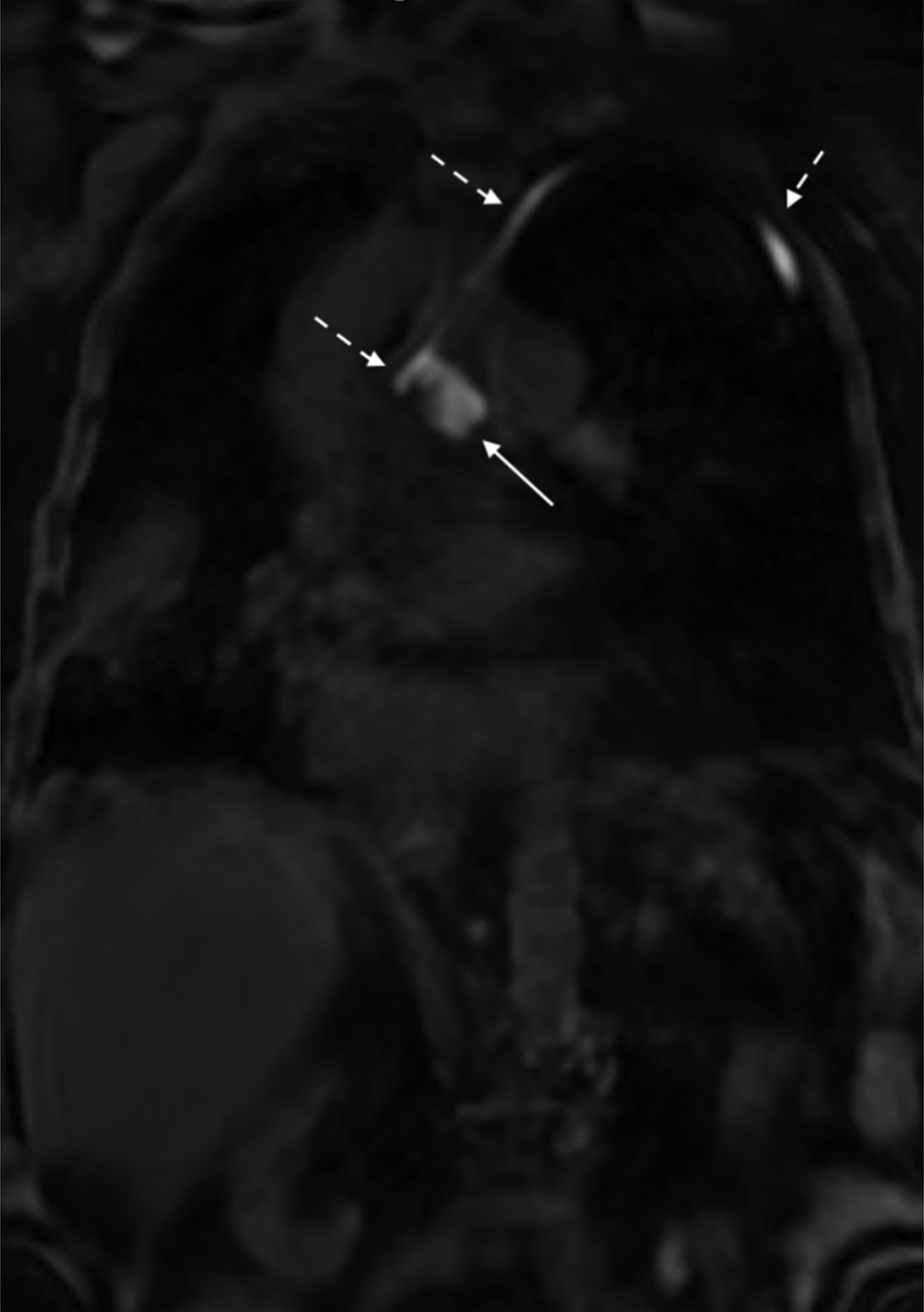
Patient after oesophagectomy with high output from mediastinal drain. The coronary T1-image showed enhancing middle mediastinum chylous collection (white arrow) with a drain *in-situ* and curving up the left apex (white broken arrows).

This is a 75-year-old male patient who also had recent oesophagectomy due to oesophageal malignancy. He had ongoing high-volume output from a mid-mediastinal drain that was left *in-situ* post-oesophagectomy. This patient had DCE MR lymphangiogram to investigate the site of chyle leak. The coronal *T*_1_ weighted images with fat saturation showed accumulation of gadolinium in the middle mediastinum, and the tip of the surgical drain is positioned in the superior aspect of the collection (Figure 5).

### Case 4

**Figure 6.**
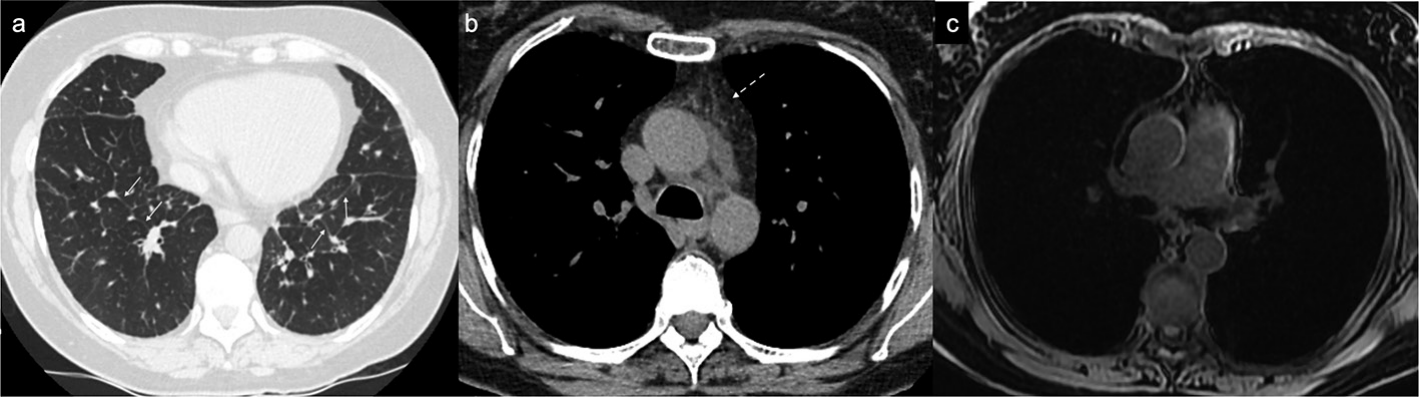
Patient present with dyspnoea and chest CT findings of mediastinal fat stranding (white broken arrow) and bilateral smooth interlobular septal thickening (white arrow) (Figure 6a–b), and raising possibility of pulmonary lymphangiomatosis. DCE MR lymphangiogram showed no abnormal intra thoracic lymphatic vessels (Figure 6c). DCE, dynamic contrast-enhanced; MR, magnetic resonance.

This was a 57-year-old female patient who presented with dyspnoea and echocardiogram excluded heart failure. The chest CT of the patient showed significant stranding in the mediastinal fat and bilateral smooth interlobular septal thickening (Figure 6a–b). The chest CT findings could represent lymphatic proliferation, which raised the possibility of pulmonary lymphangiomatosis.^9,10^ Subsequent DCE MR lymphangiogram showed no evidence of abnormal dilated intrathoracic lymphatic vessels (Figure 6c).

## Discussion

In this case series, we were able to use DCE MR lymphangiography in a number of clinical situations to provide anatomic evaluation of the central lymphatic ducts including the cisterna chyli, thoracic ducts, and to exclude variant anatomy. The findings were important in confirming or excluding chyle leak as well as to provide guidance for surgical management. In the first case of the traumatic chylothorax, the MR image spatial resolution was able to locate the level of the thoracic ductal leak to aid surgical planning. The axial images of the thorax were especially useful to identify pooling of contrast in the pleural effusion. The whole investigation was completed within 30 min including the time taken for the injection of the inguinal lymph nodes. This case illustrated that DCE MR lymphangiography was relatively fast, and it was able to overcome the limited spatial resolution of lymphoscintigraphy. DCE MRI lymphangiogram is also relatively easier to perform compared to fluoroscopic lymphangiography, in which the expertise of an interventional radiologist is often required. The second and third cases demonstrated that DCE MR lymphangiography could reliably confirm or exclude chylous effusion/collection in the mediastinum after oesophagectomy. It was able to confirm successful surgical thoracic duct ligation post-oesophagectomy, and in another case to identify the location of the thoracic ductal leak which had resulted in the mid-mediastinal collection. As a result, DCE MR lymphangiogram has become a valuable diagnostic tool in the surgical setting at our institution. In situations when the patient is claustrophobic or unstable, then a coronal *T*_1_ weighted sequence with fat saturation is the sequence of choice to reduce scanning duration. The last of the case series showed that DCE MR lymphangiogram could provide assessment of thoracic and mediastinal lymphatic anatomy to exclude pulmonary lymphangiomatosis and other diseases of the thoracic lymphatic system.^11^

## Conclusion

DCE MR lymphangiography is a radiation-free and relatively quick imaging technique with high spatial resolution for evaluation of the central lymphatic system, compared to the more traditional techniques of fluoroscopic lymphangiography and lymphoscintigraphy. The current MR image quality is also capable of locating the site of thoracic ductal leaks to guide surgical planning. We recommend the radiologist working alongside the radiographer during the scan to further optimise image quality.
